# Lessons learned from the Alberta Border Testing Pilot Program

**DOI:** 10.3389/frhs.2023.1220027

**Published:** 2023-11-24

**Authors:** Jaling Kersen, Tayler D. Scory, Oluwasefunmi Akeju, Robert G. Weaver, Lianne Barnieh, Paul E. Ronksley, Jenine Leal, Dean Blue, Erin O’Neill, David J. T. Campbell, Marcello Tonelli, Meaghan Lunney

**Affiliations:** ^1^ Department of Community Heath Sciences, University of Calgary, Calgary, AB, Canada; ^2^ Department of Medicine, University of Calgary, Calgary, AB, Canada; ^3^ Ministry of Health, Government of Alberta, Edmonton, AB, Canada

**Keywords:** pandemic, international travel, border control, quarantine, policy

## Abstract

**Background:**

During the Coronavirus disease (COVID-19) pandemic, countries implemented border control and quarantine measures to reduce transmission. The Alberta Border Testing Pilot Program (ABTPP) allowed international travellers entering Alberta to reduce their quarantine period following two negative COVID-19 tests. We evaluated participant experiences with the ABTPP and implementation.

**Method:**

We used a parallel convergent mixed-methods design to explore participant experiences through electronic web-based questionnaires (*n* = 21,089; *n* = 13,839) and semi-structured telephone interviews (*n* = 30). We evaluated implementation through three staff focus groups (*n* = 11). We analysed questionnaires using descriptive statistics and analysed interviews using inductive and deductive thematic analysis. We deductively coded focus group data using the 2009 Consolidated Framework for Implementation Research (CFIR).

**Results:**

Questionnaires indicated minimal issues with registration forms (91.7%), symptom reports (95.5%), and COVID-19 testing (95.7%). Most respondents (95.1%) expressed willingness to participate in the ABTPP again. Interviews revealed three themes related to participant experience: program efficiency, clarity of information, and requisite effort. Focus groups identified key implementation facilitators including the single health information system, strong stakeholder partnerships, and good communication across partnerships. Barriers included program complexity, implementation timeline, and evolving external context.

**Discussion:**

Participants reported high satisfaction with the ABTPP. Border testing programs should have high efficiency, require low effort, and use messaging that is clear and consistent. The effective implementation of border testing programs may be facilitated by strong leadership, adaptability, automated components, good communication, and simple technology. Learnings from participants and staff may help improve the implementation of border control programs for future pandemics or other emergencies.

**Conclusions:**

The ABTTP was a novel border control measure during the COVID-19 pandemic. Our evaluation of both participant and staff experiences demonstrated high levels of traveller satisfaction and identified areas for improvement that can inform the development of future border control measures.

## 1. Introduction

The coronavirus disease 2019 (COVID-19) has had a devastating impact on human health, well-being, and the economy (1, 2). Due to its rapid onset and high transmission rate, governments were forced to implement strict public health measures with little notice in an attempt to reduce transmission. One area especially affected was international travel (3, 4). Due to the increased risk of COVID-19 case importation through international travel (5, 6), many countries implemented border measures, including closing borders, screening programs, and post-arrival quarantine (7–10). Border control measures were necessary to regulate the movement of individuals across international borders due to the need to contain the transmission of COVID-19, prevent the importation of new variants and conserve essential healthcare resources (11–13). Border closures and surveillance programs in response to a pandemic are complex (14, 15) and their implementation and effectiveness are influenced by several factors (16, 17). Learning more about how to properly implement such programs in real-world settings is important to inform future pandemic planning (18). Here, we share the lessons learned from implementing a COVID-19 border testing program in Alberta, Canada, including a modified quarantine procedure.

In March 2020, the Government of Canada introduced a travel advisory on non-essential travel and a partial border closing as well as a mandatory 14-day quarantine period for international travellers arriving in Canada (19). On November 2, 2020, the Public Health Agency of Canada (PHAC) and the Government of Alberta (20) launched a pilot program in Alberta: the Alberta Border Testing Pilot Program (ABTPP) (20). Travellers entering Canada through the Calgary International Airport or the Sweetgrass Coutts land border crossing were eligible to participate in this voluntary program. Participants testing negative for COVID-19 on entry could reduce the mandated 14-day quarantine period if they remained asymptomatic and again tested negative for COVID-19 6 days later. The program was designed to run for 6 months or until 52,000 participants were recruited, whichever occurred first. Complete details on the program including its performance in identifying imported cases have been reported elsewhere (21).

To inform border control program design and delivery for future health emergencies, we evaluated the participant experience and implementation of the ABTPP.

## 2. Materials and methods

Full details of the study methods are described in Supplementary Material (Item S1).

### 2.1. Study design

We used a parallel mixed-methods convergent evaluation involving an online, structured questionnaire, semi-structured individual interviews, and focus group discussions. Our questionnaire and qualitative studies aligned with the Checklist for Reporting Results of Internet E-Surveys (CHERRIES) (22) and COnsolidated criteria for REporting Qualitative research (COREQ) (23) guidelines.

### 2.2. Setting

When the ABTPP was implemented in November 2020, border measures had been implemented at Canadian airports and land borders for approximately 6 months. A significant drop in international flights due to these measures and the pandemic at large had been observed globally (24), including in Canada (19). All international travellers arriving in Canada were required to register through a web-based application (ArriveCAN) (25, 26), quarantine for 14 days, and submit a daily questionnaire on COVID-19 symptoms unless they were exempt from quarantine, primarily due to their employment (27). The ABTPP was launched to trial a program that would reduce the 14-day quarantine for travellers if they tested negative for COVID-19. An overview of the ABTPP for both groups of participants is described in Figures 1A,1B. Travellers not exempt from quarantine (i.e., “non-exempt”) as well as those exempt could choose to participate; however, the program requirements were different for the two groups.

**Figure 1A:**
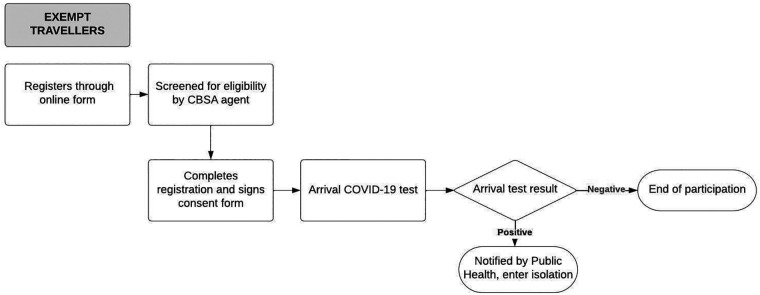
Alberta border testing pilot program pathway for exempt participants from the initiation to completion of the program. The rectangles denote processes within the ABTPP pathway, with the rounded rectangles denoting the final step of the pathway. The diamond shape denotes the arrival of the COVID-19 test result with positive or negative indicating the result of the COVID-19 test. The arrows indicate the direction in which the pathways flow. CBSA, Canadian Border Services Agency; COVID-19, coronavirus 2019.

**Figure 1B:**
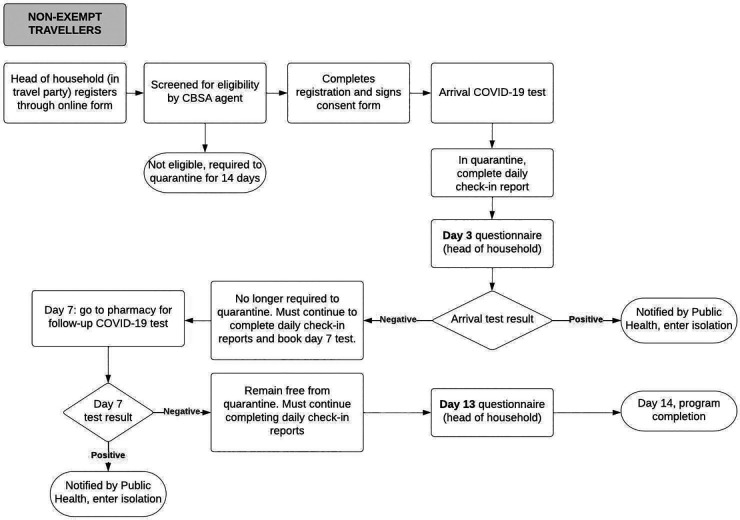
Alberta border testing pilot program pathway for non-exempt participants from the initiation to completion of the program. The rectangles denote processes within the ABTPP pathway, with the rounded rectangles denoting the final step of the pathway. The diamond shape denotes the arrival of the COVID-19 test result with positive or negative indicating the result of the COVID-19 test. The arrows indicate the direction in which the pathways flows. CBSA, Canadian Border Services Agency; COVID-19, coronavirus 2019.

Many stakeholders were involved in the design and delivery of the ABTPP, including the PHAC, Transport Canada (TC), the Government of Alberta, Alberta Health Services (AHS), and vendors contracted for logistics, customer support, and information technology (IT) services. The University of Calgary was commissioned to evaluate the program's effectiveness and implementation. The program commenced on November 2, 2020, and ran until February 21, 2021, once 52,000 travellers had participated.

### 2.3. Study participants

Two types of participants were included: (1) travellers that volunteered to participate in the ABTPP program (herein referred to as participants) and (2) program staff who were involved in the implementation of the program (herein referred to as staff). To evaluate participant experience, we used an online questionnaire and semi-structured individual interviews. To evaluate implementation, we held focus group discussions with staff.

### 2.4. Data collection

We distributed an online questionnaire to one member of each travel party on days 3 (Supplementary Table S1) and 13 (Supplementary Table S2) post-arrival, where arrival was day 1. Exempt travellers did not receive the questionnaire. If members of the same household were travelling together, one person (referred to as the “head of household”) was chosen by the travel party to complete the online registration form and the follow-up surveys.

We used purposive sampling to select a subgroup of participants based on age, gender, location of residence, and traveller type for a semi-structured interview by telephone. We interviewed both exempt and non-exempt participants after they had completed the program. A female research assistant, with training and experience conducting qualitative interviews conducted all telephone interviews, in her place of residence with no other persons present. An interview guide was utilized to facilitate the individual interviews (Supplementary Table S3).

We recruited staff involved in the program implementation separated by role (health authority, operations vendor, and traveller support) using a convenience sampling approach for focus group discussions, which were facilitated using an interview guide (Supplementary Table S4).

### 2.5. Data analysis

We analyzed quantitative data from the participant questionnaire using descriptive statistics measures of frequency (percent) and measures of central tendency (median). The qualitative data from the interviews and focus groups were analysed using different approaches. For participant interviews, we used a combination of deductive and inductive thematic analysis approaches following the approach introduced by Braun and Clarke (28). Two independent reviewers deductively coded interview transcripts to align with pre-determined phases of the program (registration, arrival test, follow-up) and inductively added codes that described the positive and negative aspects of each program phase using Nvivo (29). We deductively coded focus group transcripts following the five overarching domains within the 2009 Consolidated Framework for Implementation Research (CFIR) (30, 31) (Supplementary Table S5). The CFIR is a conceptual framework that allows for systematic evaluation of any potential barriers and facilitators of the implementation.

### 2.6. Ethics

The University of Calgary Conjoint Health Research Ethics Board (CHREB) approved this study (REB20-2147). All participants provided informed consent.

## 3. Results

### 3.1. Participant questionnaire

In total, 21,006 non-exempt participants received a link to complete the day 3 questionnaire and 20,199 (96.1%) submitted responses (Supplementary Table S1). The respondent's median age was 42 years and 54.0% were men. The median number of adults per household was 2 and most had no children at home (70.8%). 67.5% were currently employed, 7.6% were students, and 25.0% were unemployed or retired. 13,240 received a link for the day 13 questionnaire and 12,502 (94.4%) submitted responses (Supplementary Table S2). Day 13 respondents had a mean age of 43 years and 53.3% were men.

### 3.2. Day 3 questionnaire

A large majority of respondents reported no difficulties with the early phases of the program, including the online registration form (91.7%), finding the in-person registration desk (94.0%) or testing site (95.7%) at the port of entry, or completing the first 3 daily check-in reports (95.5%). In total, 7.0% of participants felt the arrival test result turnaround time was not acceptable, most of whom had not received their result by the time of their day 3 questionnaire (i.e., <72 h after arrival). Overall, 16.8% reported difficulties accessing their arrival COVID-19 results.

### 3.3. Day 13 questionnaire

**Figure 2:**
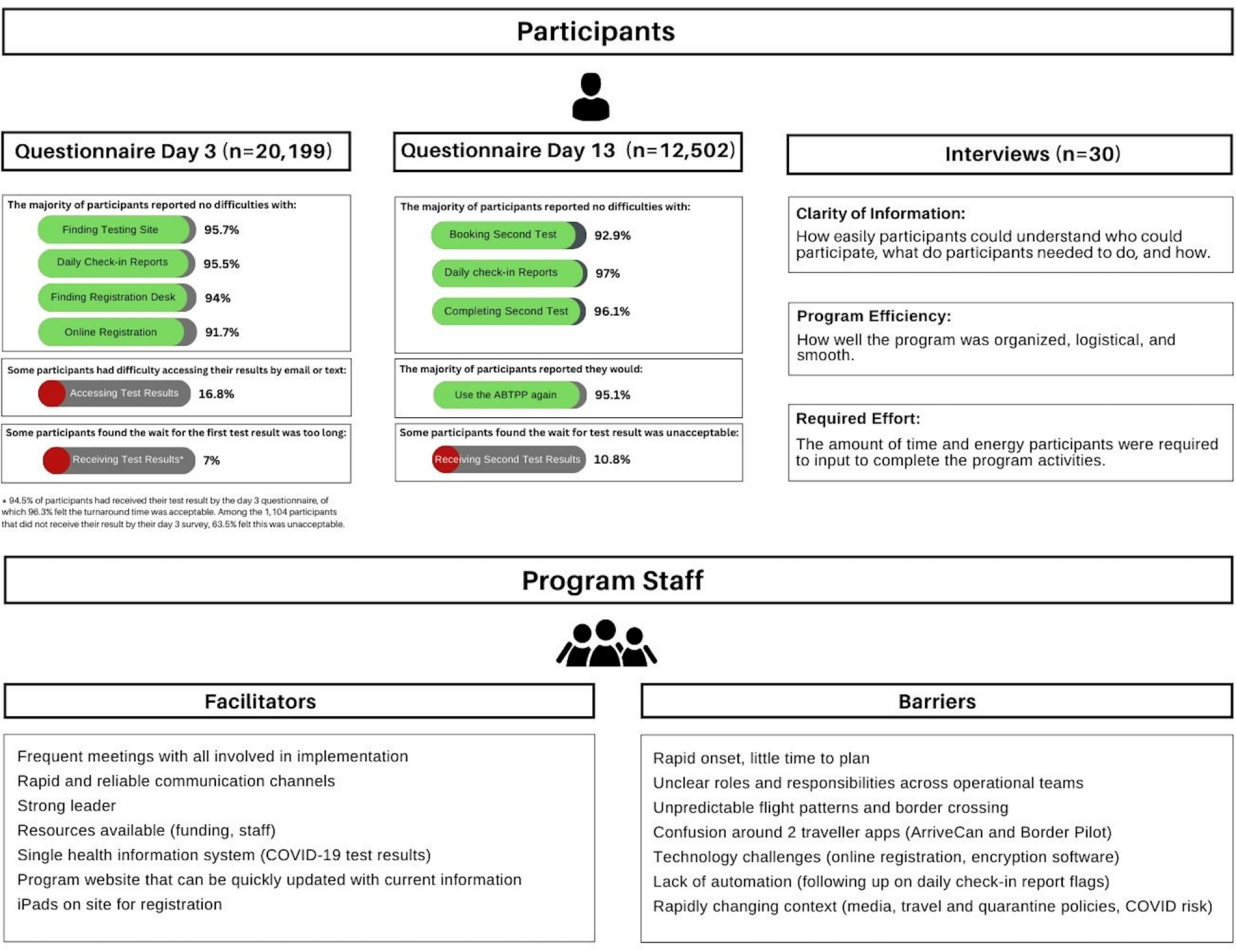
Summary of findings from participants (questionnaires and interviews) and staff (focus groups). Key findings from the quantitative (participant questionnaire) and qualitative (participant interviews and staff focus groups) methodologies. The top panel is the results of the participant questionnaires and interviews. The percentages associated with each response are listed beside with green denoting greater than 50% and red denoting less than 50%. The themes from the qualitative interviews and their definitions are listed below the interview header. The bottom panel is the facilitators and barriers identified from the staff focus groups.

Most respondents reported no difficulties booking (92.9%) or completing (96.1%) their second COVID-19 test. 10.9% of respondents felt the second test result turnaround time was not acceptable. 95.1% of respondents reported that they would use the ABTPP program again and 50.4% reported that they would not have traveled had the ABTPP been unavailable. While participants were not required to pay for COVID-19 tests as part of the program, 48.4% of respondents were willing to use the program even if required to cover costs associated with testing or administration. Of those willing to pay, 30.7% reported willingness to pay between $150 to $250 (Figure 2).

### 3.4. Participant interviews

We completed 30 semi-structured interviews with participants who arrived between December 1 and December 28, 2020. Interview participants had a median age of 41 years and 30.0% were men. 13.3% were exempt from quarantine; 83.3% were Canadian citizens or permanent residents (Table 1). Including consent and preamble, the interviews averaged 30 min each. Thematic analysis revealed 3 major factors that impacted the participant experience with the program: Clarity of Information, Program Efficiency, and Required Effort (Figure 2). Exemplar quotations for each theme are provided in Supplementary Table S6.

**Table 1:** Characteristics of ABTPP program participants who took part in the semi-structured telephone interview.

Program Participant Characteristics	*N* (%) or Median (Q1, Q3)
Number of program participants interviewed	30
Gender:	
Man	9 (30.0)
Woman	21 (70.0)
Age:	
Median (Q1, Q3)	41 (20, 70)
18 to <35	7 (23.3)
35 to <65	19 (63.3)
Over 65	4 (13.3)
Country of Residence:	
Canada	25 (83.3)
Sweden	1 (3.3)
Finland	1 (3.3)
USA	3 (10.0)
Traveller Type:	
Exempt	4 (13.3)
Non-Exempt	26 (86.7)
Point of Entry:	
Calgary International Airport (YYC)	27 (90.0)
Coutts Land Border	3 (10.0)

### 3.5. Clarity of information

Information was shared with participants about what the program entailed, who was eligible, and participation instructions. For example, the program website described eligibility and processes, a handout distributed at registration included instructions participants needed to follow, a daily email or text message containing the link to the check-in report, and COVID-19 test results and any resulting actions were also delivered by email or text. Interviews with participants indicated that the clarity of this information impacted their satisfaction with the program.

Participants who found this program information to be clear and easy to follow often described a positive experience. Specifically, some participants commented on how well staff were informed and helpful in communicating the various steps or instructions. Some expressed difficulties finding information about the program and requirements to participate*.* Others were confused by redundancies and inconsistencies in the program instructions. One participant commented on the confusion of having to submit two check-in reports across the Federal (ArriveCAN) and provincial programs, which led to anxiety.

### 3.6. Program efficiency

Second, perceptions about how smoothly the program operated were correlated with participant satisfaction. There were many steps involved in the process, including completing the online registration form, finding the program site at the airport or border crossing, providing consent, completing the arrival COVID-19 test, receiving test results, booking and completing the follow-up COVID-19 test, and submitting the daily check-in reports. Some participants described these processes as straightforward and simple and reported a positive experience.

On the contrary, participants with negative remarks described the steps of the program as inefficient, repetitive, and a waste of time. Having to wait on arrival was inconvenient for some. Others complained that the registration process was repetitive or posed technical challenges.

### 3.7. Required effort for program completion

Third, the effort required to complete the various program components also influenced participants' experience. Although some complained of long lineups and repetitive forms at arrival, many felt the amount of effort required was minimal. Similarly, many participants felt the burden of submitting the daily check-in reports was low.

To complete the second COVID-19 test, participants were required to schedule an appointment at a nearby pharmacy. Some participants felt the effort to schedule and attend these appointments was minimal. On the other hand, some participants complained that completing the second COVID-19 test was more burdensome than expected. Lastly, some participants criticized the process of receiving COVID-19 test results, especially related to the encryption software.

Overall, participants reported a positive experience, despite some inconvenience, and felt the program benefited themselves and society at large by reducing risks associated with international travel.

### 3.8. Staff focus groups

Three focus groups with staff members were conducted using Microsoft Teams on February 22 (*n* = 4), February 26 (*n* = 4), and March 2 (*n* = 3), 2021. Staff included members of AHS (group 1), a logistics and operations vendor (group 2), and a participant support vendor (group 3). Using the CFIR framework (32) (Supplementary Table S5), we identified facilitators and barriers to implementing the ABTPP. We focused on aspects of the program itself (CFIR domain: Intervention Characteristics), external factors (CFIR domain: Outer Setting), internal factors (CFIR domain: Inner Setting), and how the program was implemented (CFIR domain: Process). Exemplar quotations are provided in Supplementary Table S7.

### 3.9. Intervention characteristics

Certain aspects of the program design, including how the ABTPP was advertised and the activities and materials involved impacted implementation. Some staff felt the program could have been better presented to travellers through better communication about eligibility and what participation involved. For example, staff agreed that some of the program activities confused participants, especially related to having two sources for daily symptom reporting or receiving test results.

Similar to results from the participant interviews, staff also recognized the frustration that participants experienced when completing paperwork or receiving test results. Additionally, some staff felt that certain elements of the program were not accessible to everyone, especially people that did not speak English or French, did not have access to a charged smartphone, or lived in rural areas.

### 3.10. Outer setting

Ongoing changes related to COVID-19 transmission risk and variants, travel restrictions, flight or border-crossing patterns, pre-departure COVID-19 test requirements, and Federal and Provincial policies also created implementation challenges. Staff acknowledged how the changing external policies related to quarantine requirements were challenging to manage and required the program to rapidly adapt, often with short notice and little information. This created confusion among staff and participants regarding eligibility and testing requirements.

### 3.11. Inner setting

The existing structure of the local health authority (AHS) and its laboratory health information system were also essential for the ABTPP. Prior to program launch, AHS had a well-established and provincial IT department, which included a single health information system for COVID-19 testing data. In the focus groups, staff highlighted that the successful launch of the ABTPP was facilitated by leveraging AHS's pre-existing structure and experience.

Further, the ABTPP program was the result of partnerships across the Federal and Provincial governments, the provincial health authority, laboratory services, the airport authority, and other private vendors. Many of these stakeholders were involved in the design, deployment, and evaluation of the ABTPP. Establishing strong networks and rapid and reliable communication channels between these groups was essential for program success. Some staff highlighted the advantages that stemmed from frequent meetings and strong internal communication between leaders and their staff.

However, others (typically contracted vendors) felt communication within the networks was sometimes challenging, which negatively impacted the implementation of the program. Although discussions with core implementation leads took place, external partners were not always included. Further, not all operations staff understood how roles and responsibilities were divided across all stakeholders and suggested that a group discussion with all involved may have been useful. Challenges across these partnerships and connections were noted due to overlapping boundaries or unclear roles across the various operational teams, particularly related to activities at the airport and border crossing. One staff member described a lack of acceptance when working with other organizations. Others commented on the challenges of having to shift their typical workflow to that of the airport.

### 3.12. Process

Due to the rapid and urgent onset of the COVID-19 pandemic, the time available to prepare and plan program delivery were limited. This was especially felt by contracted vendors (software developers, logistics and customer support), who were hired shortly before launch and some only had 4–5 days to prepare.

## 4. Discussion

This evaluation identified key lessons learned from implementing a border testing program in Alberta, Canada during the first year of the COVID-19 pandemic (Figure 2). Overall, the quantitative analysis indicated that the program was well received by most participants. Over 12,000 program participants completed both questionnaires and most would use the program again, reporting few difficulties with major elements of the program. Semi-structured interviews with 30 purposively sampled participants found that information clarity, delivery efficiency, and effort required from participants impacted their satisfaction with the program. Focus groups with 11 staff members reported overall great implementation success of the ABTPP, despite its urgent and complex nature. Staff highlighted the importance of creating a strong network of stakeholders with initial and ongoing communication. A provincial health information system was also identified as a key facilitator. Challenges related to limited planning time, lack of clarity across operational teams about roles and responsibilities, and a constantly changing context were noted as key barriers.

Evaluating COVID-19 border testing programs can help inform policies and decisions for future pandemics. Just as the lessons learned from the 2003 SARS and 2009/2010 H1N1 pandemics improved Canada's capacity to respond to the COVID-19 pandemic (33–35), learnings here can further increase preparedness. Report 8 of the Auditor General of Canada to the Parliament of Canada (36) lists several recommendations for the Public Health Agency of Canada and the Canada Border Services Agency, specifically related to pandemic preparedness and border control. Our evaluation may complement the Auditor General's report by providing additional detail on how best to plan and implement modified border control measures in future pandemics or other health emergencies. Below we summarize the main lessons identified in our evaluation and provide recommendations (Table 2), which may be useful in other jurisdictions across Canada or internationally (37).

**Table 2:** Recommendations for implementing an International border testing program during a pandemic.

Recommendation	Examples
Use clear and consistent messaging	For travellers: • Create a program website that is frequently updated to provide travellers with information about eligibility and what participation involves. • Provide participants with handouts at registration with clear instructions. • Establish a helpline for travellers if they have questions. • Work with media to set appropriate participant expectations (e.g., time to receive the first test) • Involve airlines to educate travellers on the flight so they are informed and prepared for registration. For staff: • Establish rapid and reliable communication channels to confirm decisions and program processes. • Encourage frequent communication between the working group and governance teams to troubleshoot and address questions.
Define roles and responsibilities of staff involved in operations and logistics	In areas with overlapping duties (airport, land border), ensure all parties understand which teams are responsible for what decisions and activities. Designate specific rooms (areas) on-site to be used in cases of public health emergencies to avoid pop-up stations and inconveniences of running a testing clinic in an airport or at a land border crossing.
Adapt the program to changing context	Update program website and traveller handouts every time there is a relevant change. Provide a helpline for travellers to address questions about changing eligibility or program requirements.
Use resources efficiently	Use one participant app (e.g., ArriveCAN). Use electronic consent instead of paper. Partner with airports, airlines, and land border crossings to receive information about flight/border crossing predictions so that the appropriate number of staff can be scheduled for testing and operations. Employ a single and integrated laboratory system to collect and share health information (e.g., COVID-19 test results) quickly and easily. Use patient portals to share test results with participants, rather than by phone or email which requires complex encryption software.
Allow ample planning time	Establish prepositioned contracts to hire contracted vendors as early as possible.
Ensure accessibility	Use electronic consent and registration forms to allow for translation into multiple languages. Have tablets on-site for travellers that do not have access to a smartphone or tablet. Increase the use of rural pharmacies.

### 4.1. Use clear and consistent messaging

Due to the rapidly evolving nature of the COVID-19 pandemic, clear communication with travellers and also within the program implementation team was imperative for operational success. This aligns with an evaluation of a similar border testing program in Alaska that also identified communication as an essential factor for operational success (38). The ABTPP had ongoing communication channels for staff to clarify questions and discuss issues and additionally provided participants with a helpline for queries or concerns, both raised as key facilitators to program success.

Good communication with travellers participating in a program ensures that they understand who is eligible, what they need to do, and how to complete the various steps. A key finding in our evaluation was that participants were confused about certain aspects of the program. For example, who was eligible, which application they were required to use for the program (e.g., ArriveCAN vs. the program-specific app), and the process for receiving test results. While not specifically identified in our study as a key theme, Ohlsen et al. noted the importance of managing travellers' expectations (38), which can be achieved through effective communication strategies. The ABTPP created a public-facing website with information about the program, as well as a telephone support line for travellers. Additionally, involving airlines to share information about the program with travellers during the flight may help reduce confusion upon arrival. Similar communication strategies were found to be useful in the Alaskan program (38).

### 4.2. Define roles and responsibilities among operational teams

Given the large number of operational partners involved in the ABTPP, there was initial confusion regarding logistics and decision-making on-site. Instead of operating at an AHS facility, popup COVID-19 testing stations were being created at the airport and land border crossing. During the early implementation phase, there was uncertainty around how responsibilities (e.g., photocopying, ordering, and paying for supplies) or decision-making capacity (e.g., consent process, how to address significantly delayed flights) should be divided among airport and border staff, AHS, and contracted vendors. Given that healthcare, the airline industry, and border services are all independently complex, a program that combines these sectors would benefit from establishing clear roles and responsibilities among these operational teams. Additionally, establishing a designated space at these locations for public health activities, such as screening, enrollment, and testing, may reduce some of the barriers and could be considered for future pandemics.

### 4.3. Adapt the program to changing context

Throughout implementation, ABTPP administrators and staff were faced with a rapidly evolving context that impacted operations, including changes in the risk of COVID-19 transmission, flight or border crossing patterns, or restrictions on entry. As with other industries [e.g., education, business, tourism, and hospitality (39)], designing a program that is flexible and able to be adapted quickly is needed to handle the constantly evolving guidelines and policies. For example, flight patterns significantly fluctuated from November 2020 to February 2021, likely related to transmission, typical seasonal variation (e.g., holiday, vacation), and others. The staff of the ABTPP struggled with staffing, especially nurses, as it was difficult to anticipate demands due to the fluctuations in flight patterns and passenger volumes. Asking airlines to share projections about upcoming flights and passenger volumes would be helpful to inform program staffing requirements.

Changes in ABTPP eligibility due to new variants also caused traveller confusion. In December 2020, travellers arriving from certain jurisdictions (e.g., the UK) were required to quarantine and thus not eligible. Ensuring participants are clear on eligibility through a current website or helpline may reduce this confusion. Involving airlines with program delivery may allow travellers to be informed about the program's requirements while still in flight, which may streamline processes on arrival.

### 4.4. Use resources efficiently

Resource use is another consideration when planning or implementing a border testing program (38). Materials (especially personal protective equipment) are needed to disinfect the site and equipment, register and test participants, and keep participants and staff safe. On-site nurses are required to collect biological samples and other staff are required to register, follow, and communicate with participants. The ABTPP developed an entirely new web-based application to minimize workload and streamline participant registration and follow-up processes. Strategies to potentially improve program efficiency and reduce costs identified by our research included using an online registration and consent form (instead of paper to reduce disinfecting supplies and time), a comprehensive website with detailed and clear program information for participants (reducing the need for telephone support calls), using easy methods for sharing test results with participants (such as the existing patient portal) to reduce the need for laboratory personal to investigate unconfirmed results, automating forms to reduce errors and making fields editable to update records as needed, and providing operations staff with flight volumes in advance to staff accordingly. Further, although the ABTPP was publicly funded, nearly half of the participants indicated a willingness to pay to participate in the program, presumably as it offered a reduced quarantine. Cost-recovery mechanisms could be considered for future border control programs that offer a similar benefit to travellers.

Of note, Alberta was well positioned to execute mass COVID-19 testing due to its single health information system; however, evidence from other jurisdictions that lack such a system confirms the importance of strong laboratory information systems for COVID-19 prevention and control (40–42). Exploring opportunities through the Pan-Canadian Health Data Strategy (43) may help improve how data are collected and shared to ensure optimal privacy and access for travellers.

### 4.5. Allow ample planning time

During implementation, adequate time to plan is needed to design, test, and refine the program. Due to the urgency of the COVID-19 pandemic, such planning time was limited. Further, airports and border crossings are not traditional healthcare facilities, and workflow, logistical, and legal expertise outside of the traditional health authority was required. Therefore, external vendors were contracted for logistics planning, program application software development, and customer support. These contractors were given only a few days from the time the contract was awarded to launch. Establishing prepositioned contracts (44) for public health emergencies may allow vendors to begin earlier, resulting in a smoother implementation during the first few weeks of execution.

### 4.6. Ensure accessibility

Staff (especially those working in customer support) noted that some participants may have experienced greater barriers than others, especially those that do not speak English or French, do not have access to a smartphone or tablet, or live in rural areas. Having online forms that can easily be translated, tablets on-site with support to assist with registration, and rural testing areas or remote testing may improve program accessibility.

### 4.7 Limitations

The scope and applicability of our findings and recommendations emerging from this evaluation should be approached while accounting for the limitations of this study.

Firstly, this evaluation includes select perspectives on the implementation of the ABTPP. Although the program occurred from November 2, 2020, to February 21, 2021, questionnaires were only administered beginning December 13, 2020, and only respondents arriving between December 1 and December 28, 2020, were interviewed. We intentionally began collecting feedback a few weeks after the program start date to eliminate challenges related to early program initiation, which has reduced our ability to reflect on this phase of the program.

Secondly, only members of the travel party who self-identified as the “head of the household” received a questionnaire or were selected to participate in the interview and so we may have missed some perspectives. Respondents that tested positive for or re-entered quarantine for any other reason did not receive the 13-day questionnaire and a technical error resulted in missing day 13 questionnaires from all travellers arriving between December 13, 2020, and January 1, 2021 (a total of 7,676 missed responses).

Thirdly, while focus groups offer a dynamic group interaction, there may be limitations if participants have an existing relationship. For example, staff may be less willing to share ideas if their supervisors are present or they are concerned their opinions or participation may negatively impact their employment. Further, not all personnel involved in implementation participated in the focus groups and important perspectives may have been missed.

Fourthly, our intention was to evaluate the implementation of the ABTPP throughout its entire duration. We included participant perspectives throughout; however, staff may have potentially reflected on the challenges during the initial launch, which could have over-emphasized barriers.

Lastly, perspectives may have differed systematically between certain sub-groups (e.g., business travellers vs. families; younger vs. older travellers). However, as this study was not powered to look at subgroups, we were unable to determine whether any discordances indeed existed.

## 5. Conclusions

Our evaluation of the ABTPP participant experience and implementation demonstrates a high degree of traveller satisfaction and indicates that most respondents were willing to volunteer for the program again during its implementation period. To ensure participant satisfaction, border testing programs should be efficient, require low effort from participants, and use clear and consistent messaging. Strong leadership, good communication across networks (including contracted vendors), adaptability, automated components, and simple technology may facilitate effective implementation. These lessons learned may help to inform border control measures during future pandemics or other emergencies.

## Data Availability

The datasets presented in this article are not readily available due to privacy concerns. Requests to access the datasets should be directed to; cello@ucalgary.ca.
