# Supplementary material for: Lessons learned from the Alberta Border Testing Pilot Program
Source: Front Health Serv. 2023 Nov 24;3:1220027. doi: 10.3389/frhs.2023.1220027 (PMC10704600; doi:10.3389/frhs.2023.1220027)
Supplement: Supplementary file 1 [file Datasheet1.docx]

Supplementary Material

**Lessons learned from the Alberta Border COVID-19 Testing Pilot Program: an implementation evaluation**

**Jaling Kersen , Tayler D Scory, Oluwasefunmi Akeju, Robert G Weaver, Lianne Barnieh, Paul E Ronksley, Jenine Leal, Dean Blue, Erin O’Neill, David JT Campbell, Marcello Tonelli*, Meaghan Lunney**

*** Correspondence:**
Dr. Marcello Tonelli

[cello@ucalgary.ca](mailto:cello@ucalgary.ca)

403-220-7906

2500 University Drive N.W Calgary, Alberta, Canada, T2N 1N4

# Supplementary Data

**Supplemental Item S1. Methods**

*Study Design*

We used a mixed methods approach (parallel convergence) to evaluate the ABTPP, including an online questionnaire, individual interviews, and focus group discussions. To learn the traveller's perspective, we distributed an electronic closed-ended survey and individually interviewed participants by telephone. To learn the staff perspective, we held online focus group discussions. Our survey and qualitative studies aligned with the CHERRIES [1] and COREQ guidelines [2], respectively.

*Setting*

At the time the ABTPP was implemented in November 2020, border measures had been implemented at Canadian airports and land borders for approximately 6 months. A significant drop in international flights due to border closures and the pandemic at large had been observed globally[3], including in Canada[4]. All international travellers arriving in Canada were required to register through a web-based application (ArriveCAN)[5, 6], quarantine for 14 days, and submit a daily questionnaire on COVID-19 symptoms unless they were exempt from quarantine primarily due to the nature of their employment[7].

On November 2, 2020, a partnership between the Public Health Agency of Canada (PHAC) and the Government of Alberta launched the ABTPP at the Calgary International Airport (YYC) and the Coutts Land Border. Many stakeholders were involved in the design and delivery of the program, including the PHAC, Transport Canada, the Alberta government, Alberta Health Services, and external vendors who were contracted for logistical, customer support, and Information Technology (IT) services. The University of Calgary was commissioned to evaluate the program’s effectiveness and implementation. The ABTPP operated until February 21, 2021, at which time it was suspended due to the launch of a Federal arrival testing program, beginning that same date. This evaluation includes travellers and staff that were involved in the program between November 2020 to February 2021.

*Study participants*

Two types of participants were included in this study: (1) travellers that participated in the ABTPP program (herein referred to as participants) and (2) staff that were involved in the implementation of the program (herein referred to as staff). Both the non-exempt and exempt process was evaluated. However, the focus of the evaluation was on the non-exempt experience as this comprised most participants and the exempt process was a less extensive version of the non-exempt program (See Figure 1 in the article).

*Data collection*

Participant questionnaire

A questionnaire to measure participant satisfaction with the program was distributed to non-exempt participants along with their daily check-in report on days 3 (Supplemental Table S1) and 13 (Supplemental Table S2) of their program, where arrival is day 1. The questionnaire was distributed using the same method (text, email, or phone call) as the daily check-in report, which was selected by the participants during registration. Responses submitted by text or email were automatically captured into the program database. Respondents that selected phone received a phone call from staff, and responses were manually entered by the staff member.

If members of the same household were traveling together, one person (referred to as the ‘head of household’) was chosen by the travel party to complete the online registration form and the follow-up surveys. This minimized the burden on other travellers and using the household as the unit of analysis was deemed sufficient to evaluate the participant experience of the ABTPP. The ‘head of the travel party’ was selected to minimize the burden on the respondent’s time while participating in the ABTPP. The questionnaire was not public-facing and only available to this sub-group of participants. As the questionnaire was part of the daily check-in and required activity of the program, we did not advertise or promote the questionnaire nor offer incentives for completion. Respondents were not able to submit partially completed questionnaires, therefore only completed questionnaires were included for analysis.

The day 3 questionnaire collected respondent demographics along with perspectives regarding the difficulty and acceptability of program components related to registration or the arrival test at the airport or border. The day 13 questionnaire was collected only if the participants were still active in the program by day 13 (i.e., had not tested positive for COVID-19 or had not withdrawn for any other reason) and measured satisfaction with follow-up processes. The day 13 questionnaire asked about respondent difficulty with the follow-up processes (daily check-in reports, booking the second test, etc.) along with a willingness to use again and other indicators of respondent satisfaction with the program.

As travellers registered to participate in the ABTPP, they were informed that participation also required completing the satisfaction questionnaires as part of the program evaluation. Questionnaire administration and data protection were managed by the developer contracted to build and execute the program platform. The research team designed the questions.

Questionnaire items were not randomized and were all presented on one page. Some logic was incorporated so that only relevant questions appeared. For example, if a respondent indicated on the day 13 questionnaire that they would not have traveled had the program not been available, we did not ask if they would have missed work and whether the income would have been lost as a result of this missed work. All items that were not conditional on a prior item were mandatory and flagged if skipped. Participants were unable to submit until all required questions were answered. Radio buttons were used to ensure only one response per item. Respondents were able to review their answers (as the questionnaire was only on one page) and could change them before submitting. Once the questionnaire was submitted with their daily check-in, respondents could no longer use the link to repeat the survey. Members of the research team tested the usability of the questionnaires prior to launch.

Individual participant interviews

Program participants, both exempt (i.e., not required to quarantine due to a number of reasons including providing essential services, transported goods, etc.) and non-exempt travellers were eligible to participate in the semi-structured interviews. We purposively selected a cohort of 200 people enrolled in the program using maximum variation sampling of pre-specified characteristics (age, gender, location of residence, and traveller type [exempt vs. non-exempt]) to ensure we capture a wide range of perspectives. A female research assistant (JK) conducted all telephone interviews, in her place of residence with no other persons present. Participants were contacted in ascending order of their randomly assigned program ID between February 17 2021 to March 3, 2021. JK conducted the interviews following an interview guide, which had built-in prompts and had been pilot-tested (Table S1). JK was trained in qualitative methods, ethics, and confidentiality and had experience conducting qualitative interviews. JK did not have a prior relationship with any of the participants. The research assistant attempted to reach the traveller on three separate occasions, if the traveller was unavailable or declined to participate, they were not further contacted.

Participants were provided with the purpose of the study and some characteristics of the interviewer such as their name, role, and credentials. Participants were told that participation was voluntary and what would be involved, should they choose to participate. Only participants that provided verbal consent were interviewed. Including preamble and consent, the semi-structured interviews were approximately 30 minutes in length and were not repeated. All individual semi-structured interviews were audio-recorded and transcribed to ensure accurate documentation. Participant demographic information and interview responses were collected and managed using the Research Electronic Data Capture (REDCap) platform. REDCap is a secure software platform that supports data capture for research by providing 1) an intuitive interface for validated data entry; 2) audit trails for tracking data manipulation and export procedures; 3) automated export procedures for seamless data downloads to common statistical packages and 4) procedures for importing data from external sources. Once demographic and thematic saturation (i.e., no novel themes emerged with additional interviews) were achieved, the interviews were halted.

Staff focus groups

We recruited staff using a convenience sampling approach including those across multiple roles (operations, information technology, traveller support, etc.). We invited staff from Alberta Health, Alberta Health Services, and two external vendors to participate via email. Three focus groups were completed, all facilitated by a female Research Associate (ML), who has extensive experience conducting qualitative methods and was trained in qualitative methods, ethics, and confidentiality. ML did have a professional relationship with some of the participants before the semi-structured interview; however, participation was voluntary and confidential.

Participants were provided with the purpose of the study and some characteristics of the interviewer such as their name, role, and credentials. The focus groups were approximately 1 hour each in length. All focus groups were conducted over videoconference (Microsoft Teams). ML followed a semi-structured interview guide (Supplemental Table S3). The facilitator and note-taker reviewed the guide in preparation of the discussions but did not pilot test with staff. JK was present at two of the focus groups as the only nonparticipant and field notes were taken for all focus groups either by JK or ML. All focus groups were audio-recorded, then anonymized and transcribed by a professional transcriptionist to minimize any loss and ensure accurate documentation. During transcription, any personal identifiers were removed.

*Data analysis*

Questionnaire

Data from the day 3 and 13 questionnaires were analyzed using descriptive statistics. All items were presented as the number and proportion of respondents. The respondent age, number of adults and children in the household, and number of missed work days were also presented as a median with 25^th^ and 75^th^ percentile*.* Questionnaire results are presented in Supplemental Tables S1 and S2.

Participant Interviews

A total of 72 program participants were contacted by telephone to invite them to participate in the interview; 42 declined. In total, we completed 30 semi-structured interviews with participants in the ABTPP. We used a combination of deductive and inductive approaches to thematic analysis to analyze the participant interview transcripts. We deductively coded the transcripts to align with pre-determined phases of the program (e.g., registration, COVID-19 testing, check-in reports, etc.) and inductively assigned themes describing the participant experiences for each of the program phases. Our analysis followed Braun and Clarke’s approach [8] to thematic analysis which consists of 6 steps which include familiarization, coding, searching for themes, reviewing themes, defining themes, and writing. Thematic analysis was facilitated by the qualitative software NVivo[9]. Two coders (JK and ML) independently coded the data using NVivo[9] and met regularly (every 10 interviews) to review and compare emergent themes related to program aspects that participants experienced as positive, negative, or neutral. Exemplar quotes for the themes were presented in the supplemental material (Supplemental Table S6). The transcripts or analyses were not returned to the participants for review.

Focus Groups

The same two members of the research team (JK, ML) independently coded the focus group transcripts using NVivo (19) and met after coding each transcript to compare and organize themes. JK and ML deductively coded the transcripts following the constructs and domains of the 2009 Consolidated Framework for Implementation Research (CFIR)[10] which is a conceptual framework that allows for systematic evaluation of any potential barriers and facilitators of the implementation.

The 2009 CFIR is composed of five overarching domains that may affect the implementation of the intervention^16^. These five domains are Intervention Characteristics, Outer Setting, Inner Setting, Characteristics of Individuals, and Process. Intervention Characteristics are defined as components of the intervention that may influence its implementation. There are eight constructs within the Intervention Characteristics domain. The Outer setting is defined as the components of the intervention that may influence the implementation. There are four constructs in the Outer Setting domain. The Inner Setting includes components of the implementing organization that may affect the implementation of the intervention. There are twelve constructs within the Inner Setting domain. Characteristics of Individuals are defined as an individual's features or characteristics that may influence the implementation of the intervention. There are five constructs in this domain. Lastly, Process includes strategies or tactics that can influence the intervention implementation. There are eight constructs within this domain.

JK and ML evaluated, discussed and agreed on a valence rating for each construct using the valence rating system developed by the author of the CFIR [11] (Supplemental Table S5). This valence rating system ranges from -2 to +2 and uses the coded data to evaluate the influence each construct had on the implementation of the ABTPP; whether it facilitates (+) or hinders (-) the implementation and to what magnitude. The valence rating for a construct is 0 if it has a neutral effect (i.e., there is no positive or negative influence upon the implementation). An * indicates mixed opinions among respondents (some felt it was a facilitator, others felt it was a barrier). A ‘missing’ value is assigned for CFIR constructs that did not have any associated coded data. The valence ratings are presented in the supplemental material (Table S5). Exemplar quotes for each construct that highly facilitated or hindered the ABTPP are presented in the supplemental material (Supplemental Table S7). The transcripts or analyses were not returned to the participants for review.

**References**

1. Eysenbach G. Improving the Quality of Web Surveys: The Checklist for Reporting Results of Internet E-Surveys (CHERRIES). J Med Internet Res. 2004;6(3):e34. doi: [10.2196/jmir.6.3.e34](https://doi.org/10.2196%2Fjmir.6.3.e34).

2. Tong A, Sainsbury P, Craig J. Consolidated criteria for reporting qualitative research (COREQ): a 32-item checklist for interviews and focus groups. Int J Qual Health Care. 2007;19(6):349-357. doi: [10.1093/intqhc/mzm042](https://doi.org/10.1093/intqhc/mzm042).

3. International Civil Aviation Organization. 2020 passenger totals drop 60 percent as COVID-19 assault on international mobility continues (2020).<https://www.icao.int/Newsroom/Pages/2020-passenger-totals-drop-60-percent-as-COVID19-assault-on-international-mobility-continues.aspx>. [Accessed 29 August 2022].

4. Statistics Canada. Crossing the border during the pandemic: 2020 in review (2021). <https://www150.statcan.gc.ca/n1/pub/45-28-0001/2021001/article/00007-eng.html>. [Accessed 29 august 2022].

5. Government of Canada. Use ArriveCAN to enter Canada (2022). <https://www.canada.ca/en/public-health/services/diseases/coronavirus-disease-covid-19/arrivecan.html>. [Accessed 10 march 2022].

6. Government of Canada. Government extends international travel restrictions (2020). <https://www.canada.ca/en/public-safety-canada/news/2020/10/government-extends-international-travel-restrictions.html>. [Accessed 10 march 2022].

7. Government of Canada. Quarantine Exemptions for Essential Workers (2021). <https://www.publicsafety.gc.ca/cnt/trnsprnc/brfng-mtrls/prlmntry-bndrs/20210907/10-en.aspx>. [Accessed 15 march 2022].

8. Clarke V, Braun V, Hayfield N. Thematic analysis*. Qualitative psychology: A practical guide to research methods.* 2015;222:248.

9. QSR International Pty Ltd. NVivo. Version 12 [software]. 20 March 2018 [cited 15 march 2022]. Available from:<https://www.qsrinternational.com/nvivo-qualitative-data-analysis-software/home>.

10. CFIR. The Consolidated Framework for Implementation Research, <https://cfirguide.org/>; 2022 [Acessed 1 june 2022].

11. Damschroder LJ, Lowery JC. Evaluation of a large-scale weight management program using the consolidated framework for implementation research (CFIR). Implement Sci: 2013;8(1):51. [doi:10.1007/s13142-016-0424-6](https://doi.org/10.1007/s13142-016-0424-6).

# Supplementary Figures and Tables

**Supplemental Table S1: Respondent characteristics and reported satisfaction and experience with the program (Day 3).^1^**

| **Questionnaire item** | **N (%) or Median (Q1, Q3)^2^** |
| --- | --- |
| ***Day 3 questionnaire*** | |
| Number of participants that received a survey | 21,006 |
| Number of respondents that completed a survey (response rate) | 20,199 (96.1%) |
| Age of respondents:       Median (Q1, Q3)       Under 18       18 to < 35       35 to < 65       Over 65 | 42 (31, 55)  47 (0.2)  6,694 (33.1)  11,776 (58.3)  1,682 (8.3) |
| Gender of respondents:       Man       Woman       Other       Undisclosed | 10,898 (54.0)  9,230 (45.7)  25 (0.1)  46 (0.2) |
| Number of adults living in the household (including travellers):       Median (Q1, Q3)       1       2       3       4 or more | 2 (1, 2)  5,999 (29.7)  9,777 (48.4)  2,614 (12.9)  1,809 (9.0) |
| Number of children (<18 years) living in the household:       Median (Q1, Q3)       0       1       2       3 or more | 0 (0, 1)  14,300 (70.8)  2,374 (11.8)  2,336 (11.6)  1,189 (5.9) |
| Current employment status:        Employed [including self-employed]        Unemployed        Student        Retired | 13,629 (67.5)  2,090 (10.3)  1,534 (7.6)  2,946 (14.6) |
| If employed, industry breakdown:        Management occupations        Business, Finance and Administration        Natural and applied sciences and related occupations        Health occupations        Occupations in education, law and, social, community and government services        Occupations in art, culture, recreation and sport        Sales and service occupations        Trades, transport and equipment operators and related occupations        Natural resources, agriculture and related production occupations        Occupations in manufacturing and utilities        Other | 1,521 (11.2)  2,010 (14.7)  381 (2.8)  900 (6.6)  1,087 (8.0)    411 (3.0)  1,617 (11.9)  1,307 (9.6)  1,337 (9.8)  355 (2.6)  2,703 (19.8) |
| Did you have difficulties completing the online form to register for this program?        No difficulties        Some difficulties        A lot of difficulties | 18,522 (91.7)  1,474 (7.3)  203 (1.0) |
| Once you submitted the online form, did you have difficulties receiving your program Confirmation ID?        No difficulties        Some difficulties        A lot of difficulties | 19,257 (95.3)  771 (3.8)  171 (0.8) |
| When you arrived to the airport or Coutts border, you were given a program instruction sheet (purple writing). Was the information provided clear?        Yes        No | 19,373 (95.9)  826 (4.1) |
| Did you have difficulties finding the registration desk at the airport or Coutts border to complete your enrolment for this program?        No difficulties        Some difficulties        A lot of difficulties | 18,995 (94.0)  1,115 (5.5)  89 (0.4) |
| Once you found the desk, did you feel the length of time it took to complete your enrolment was acceptable?         Yes         No         Missing | 18,689 (92.5)  1,479 (7.3)  31 (0.2) |
| Did you have difficulties locating the testing station to receive your COVID-19 test at the airport or Coutts border?        No difficulties        Some difficulties        A lot of difficulties | 19,330 (95.7)  793 (3.9)  76 (0.4) |
| Have you received the result from the COVID-19 test you took at the airport or Coutts border?         Yes         No | 19,095 (94.5)  1,104 (5.5) |
| <If Yes> Did you feel the length of time it took to get your test result was acceptable?         Yes         No | 18,390 (96.3)  705 (3.7) |
| <If No> Is the length of time it is taking you to get your test result acceptable?         Yes         No | 403 (36.5)  701 (63.5) |
| Did you have difficulties accessing your test result through email or text (problems with encryption or other)?        No difficulties        Some difficulties        A lot of difficulties        Missing | 15,712 (77.8)  2,747 (13.6)  638 (3.2)  1,102 (5.5) |
| The program requires you to submit a check-in report each day about symptoms. Have you had difficulties completing these questions?        No difficulties        Some difficulties        A lot of difficulties | 19,287 (95.5)  795 (3.9)  117 (0.6) |
| If you contacted the eHealth team for support, did you find this helpful?        Yes        No        Not applicable (I have not contacted the eHealth team) | 1,968 (9.7)  411 (2.0)  17,820 (88.2) |

^1^ Among non-exempt travellers who completed the enrolment form on behalf of the family/travel party (i.e., head of household) arriving on or after December 13, 2020.

# Q1 = first quartile (25th percentile), Q3 = third quartile (75th percentile).

**Supplemental Table S2: Respondent characteristics and reported satisfaction and experience with the program (Day 13).^1^**

| **Questionnaire item** | **N (%) or Median (Q1, Q3)^2^** |
| --- | --- |
| ***Day 13 questionnaire*** | |
| Number of participants that received a survey^3^ | 13,240 |
| Number of respondents that completed a survey (response rate) | 12,502 (94.4%) |
| Age of respondents:       Median (Q1, Q3)       Under 18       18 to < 35       35 to < 65       Over 65 | 43 (32, 56)  27 (0.2)  3,846 (30.8)  7,468 (59.7)  1,161 (9.3) |
| Gender of respondents:        Man        Women        Other        Undisclosed | 6,666 (53.3)  5,794 (46.3)  16 (0.1)  26 (0.2) |
| Have you had difficulties submitting your daily check-in report about symptoms?        No difficulties        Some difficulties        A lot of difficulties | 12,128 (97.0)  336 (2.7)  38 (0.3) |
| Did you have difficulties downloading the AB TraceTogether app?        No difficulties        Some difficulties        A lot of difficulties | 11,325 (90.6)  766 (6.1)  411 (3.3) |
| Did you have difficulties booking your second COVID-19 test (swab)?        No difficulties        Some difficulties        A lot of difficulties | 11,617 (92.9)  735 (5.9)  150 (1.2) |
| Did you have difficulties completing your second COVID-19 test (swab)?        No difficulties        Some difficulties        A lot of difficulties | 12,020 (96.1)  338 (2.7)  144 (1.2) |
| Did you feel the length of time it took to get your second COVID-19 test result was acceptable?        Yes        No | 11,146 (89.2)  1,356 (10.8) |
| If you contacted the eHealth team for support, did you find this helpful?        Yes        No        Not applicable (I have not contacted the eHealth team) | 1,583 (12.7)  365 (2.9)  10,554 (84.4) |
| If this program were not available and you were required to quarantine for the full 14 days, would you have traveled?        Yes        No | 6,197 (49.6)  6,305 (50.4) |
| <If Yes> Would you have missed work?        Yes        No | 1,949 (31.5)  4,248 (68.5) |
| <If yes> Would you have had to use: (check all that apply)^4^        Vacation days (number of travellers that checked box)        Sick days (number of travellers that checked box)        Leave without pay (number of travellers that checked box)        Other (number of travellers that checked box) | 519 (N/A)  119 (N/A)  1,047 (N/A)  461 (N/A) |
| <If yes> How many days? (Median [Q1, Q3])       Vacation days:        Sick days:        Leave without pay:        Other: | 10 (7, 14)  5 (3, 12)  14 (7, 14)  14 (7, 14) |
| For future travel, would you use this program again?        Yes        No | 11,895 (95.1)  607 (4.9) |
| Would you be willing to use this program if there were fees to cover the costs for testing or program administration?        Yes        No | 6,053 (48.4)  6,449 (51.6) |
| <If Yes> How much would you pay per person?      $100 or less      $150      $200      $250 | 4,189 (69.2)  1,249 (20.6)  317 (5.2)  298 (4.9) |

^1^ Among non-exempt travellers who completed the enrolment form on behalf of the family/travel party (i.e., head of household) arriving on or after January 2, 2021. Participants who had tested positive or withdrew from the program for any other reason prior to day 13 did not receive this questionnaire.  

^2^ Q1 = first quartile (25^th^ percentile), Q3 = third quartile (75^th^ percentile).

^3^ Due to a technical glitch, 7,676 participants (non-exempt, head of household, not withdrawn) arriving between December 11 2020 and January 1 2022 did not receive a Day 13 survey.

^4^ Some participants indicated more than one type of leave from work, thus the numbers in this category are not mutually exclusive.

**Supplemental Table S3: Semi-structured telephone interview guide for program participants**

| **Interview questions:** |
| --- |
| 1. Why did you enroll in this program? |
| 1. How did you find out about the program? |
| 1. I want to start by asking about your experience with registering for the program and completing all the steps at the airport/border. Thinking of everything from filling out the registration form, to finding the program desks at the airport/border, to getting your swab, what was your experience?     Prompts:       Was it difficult to find the registration desks?       Was the information clear?       Did you have to wait a long time in line?       Were your questions addressed properly? |
| 1. Once you got to your place of quarantine, what was your experience with the rest of the program? Waiting for and receiving your first test result, booking your second, and filling out the daily check-ins?     Prompts:       Was it difficult to receive your first test result?       Were you able to easily book and get your second covid test?       Did you have any problems completing the check-in reports?       Did you know about the support team and did you ever contact them? |
| 1. Would you register for this program again for future travel? |
| 1. Do you have any final comments before we wrap up? |

**Supplemental Table S5: CFIR Valence Ratings for the Focus Groups**

| **Domain** | | **Rating** | | |
| --- | --- | --- | --- | --- |
|  |  | **Group 1** | **Group 2** | **Group 3** |
| **Intervention Characteristics** | | | | |
| Innovation source | | Missing | Missing | Missing |
| Evidence strength and quality | | -1 | Missing | Missing |
| Relative advantage | | Missing | Missing | Missing |
| Adaptability | | +1 | -1* | -1 |
| Trialability | | Missing | Missing | Missing |
| Complexity | | -1 | -2 | -2 |
| Design quality and packaging | | -1 | -2 | -2 |
| Cost | | -2 | -1 | -1 |
| **Outer Setting** | |  |  |  |
| Participant needs and resources | | Missing | -1 | -1 |
| Cosmopolitanism | | -1 | -2 | -1 |
| Peer pressure | | Missing | Missing | Missing |
| External policy and incentives | | -2 | -2 | -2 |
| **Inner Setting** |  |  |  |  |
| Structural characteristics | | +2 | Missing | +1 |
| Networks and communications | | 2* | -2 | -2 |
| Culture | | 1* | Missing | Missing |
| Implementation Climate | |  |  |  |
| Tension for Change | | Missing | Missing | Missing |
| Compatibility | | -1 | -1 | Missing |
| Relative Priority | | +1 | Missing | Missing |
| Organizational Incentives & Rewards | | Missing | Missing | Missing |
| Goals & Feedback | | Missing | Missing | Missing |
| Learning Climate | | 2* | -1 | Missing |
| Readiness for Implementation | |  |  |  |
| Leadership Engagement | | +2 | -1 | Missing |
| Available Resources | | 2* | -2 | -1 |
| Access to Knowledge & Information | | -1 | -1 | -1 |
| **Characteristics of Individuals** | |  |  |  |
| Knowledge and Beliefs about the Innovation | | Missing | Missing | Missing |
| Self-efficacy | | Missing | Missing | Missing |
| Individual Stage of Change | | Missing | Missing | Missing |
| Individual Identification with Organization | | Missing | Missing | Missing |
| Other Personal Attributes | | Missing | Missing | Missing |
| **Process** |  |  |  |  |
| Planning | | -2 | -2 | -2 |
| Engaging | |  |  |  |
| Opinion Leaders | | Missing | Missing | Missing |
| Formally Appointed Internal Implementation         Leaders | | -1 | -1 | -1 |
| Champions | | Missing | Missing | Missing |
| External Change Agents | | Missing | +1 | Missing |
| Executing | | 1* | -1 | -1 |
| Reflecting and Evaluating | | +2 | Missing | Missing |

The valence rating system ranges from -2 to +2 and uses the coded data to evaluate the influence each construct had on the implementation of the ABTPP; whether it facilitates (+) or hinders (-) the implementation and to what magnitude. The valence rating for a construct is 0 if it has a neutral effect, interviewees have mixed opinions, or there is no positive or negative influence upon the implementation. An asterisk (*) indicates that there were both positive and negative influences, but one valence rating was assigned. A ‘missing’ value is assigned for CFIR constructs that do not have coded data associated.

**Supplemental Table S6: Exemplar quotations for themes from semi-structured interviews with program participants**

| **Clarity of information** |
| --- |
| *High clarity of information* |
| “It [Alberta Border Pilot Program] was very good, it was simple to follow and they had the process down pretty well, like we knew where we were going, what the next steps were and it was quite painless.” (Participant 8) |
| “It [Alberta Border Pilot Program] was great, they were very organized. Everybody was very helpful. They explained exactly what was going to happen and when it happens and had arrows set up for everybody to follow along and it was, I thought it was a great experience,” (Participant 15). |
| “So, I didn’t complete my check-in report that day and I did receive a phone call [from the customer support team] asking why I hadn’t checked in and the staff on that phone call explained very clearly that I wasn’t out of the program yet, that I still had to do a daily check-in...” (Participant 16) |
| “[The customer support team] did an excellent job of any kind of communication. Whether it was applying for the program or the forms, it was crystal clear and everything like that”. (Participant 11) |
| *Low clarity of information* |
| “I ended up talking to I think three different people through check-ins and when they were reviewing my file and my situation and there wasn’t a lot of consistency between the people I spoke with. It just seemed like they are on different pages. I was told kind of different information, different guidelines depending on who I talked to. The person that ended up dealing with me the most at the end was really good and she was really knowledgeable and very easy to deal with, but between her and the other two people I dealt with there was a lot, I was told different things. I guess a bit more consistency would have been nice,” (Participant 16). |
| “There was a [registration] form I should have filled in and I was told that I was not in compliance…I did look up to see what the requirements there were for Canadians coming back to Canada and I did not see any mention of that form,” (Participant 26). |
| “For the check-in process…you select the person you are reporting for. Then, after you select that person, you are asked to give the birthdate, and with five people in my family…when I went to the next page, and I wasn’t sure I was putting in the right birthdate”. (Participant 16) |
| “I was explained that until I get my result I would have to check in … but, there were two places I was getting the request to check in, so I was getting a text with a reminder that I have to go online and report my health and that there was also an app that was sending me reminders about that… which was very annoying because I was all the time scared that I would fill out one of them and I wasn’t sure which one I already filled. So, there are two different places that you have to report to. I don’t know why they could not be combined into one,” (Participant 29). |
| “I think the thing that I was kind of really dissatisfied about was I don’t understand my results. When my results came in there wasn’t enough information. Like it just said, positive or negative… I wish I could have known more, more details of the test”. (Participant 28) |
| “I found I got a little mixed up between the Canada arrives system and the Alberta pilot. That took a little extra time to make sure I had all the information everybody needed. (Participant 14). |
| **Program efficiency** |
| *High program efficiency* |
| “Everything at the airport was pretty easy. It was quick. It was all pretty convenient,” (Participant 30). |
| “It was all really straightforward. Really easy, I never had any problems at all,” (Participant 06). |
| “It was very simple, very quick, very simple, very efficient,” (Participant 07). |
| “It was very good, it was simple to follow and they had the process down pretty well, like we knew where we were going, what the next steps were and it was quite painless,” (Participant 08). |
| “I mean it took mere moments in the grand scheme of things. Easy, painless,” (Participant 27). |
| “The experience was very good and just I didn’t have to wait for long and yeah everything went very smoothly, and the process was very easy,” (Participant 14). |
| “Everything about the [COVID-19] test is easy. Everything is simple, easy to understand, no problem,” (Participant 03). |
| *Low program efficiency* |
| “I mean the challenge, not the challenge but the annoying part is, you fill out the online registration and then when you get to the testing site, you know you still have to fill out more paperwork, i.e., the consent. Well, I registered for the program, and I have provided the access code. I don’t understand why I need further paper consent to participate,” (Participant 05). |
| “I think I was in three lineups. You lineup for this. Do something there. Lineup for that. Do something there, and then go to the final lineup where they took the sample. It was, I think needlessly time consuming,” (Participant 26). |
| “[The program was] frustrating … it seemed like I stood in three different lines, answered the same three questions over and over”. (Participant 28) |
| “It was a little hectic actually. We, I did the online already and it kind of crashed a couple of times… When we came back we had to go through a lot of lines to actually get to where the testing site is and we had to fill out quite a bit of forms again. It takes quite a long time,” (Participant 02). |
| **Effort required by participants** |
| *Minimal effort* |
| “The daily check-ins via email were really easy to complete,” (Participant 09). |
| “[The ABTPP processes] at the airport was pretty easy. It was quick. It was all like pretty convenient,” (Participant 30). |
| “I just called one of the nearest [pharmacies] and the booking was very easy and, yeah, just everything went smoothly”. (Participant 14) |
| “We were already set up with [the pharmacy]. So, we just said we’ve got to come in and we just bring in the pink piece of paper and again we just book a time and again, easy,” (Participant 03). |
| *High effort* |
| “My [test] results were communicated by email using a secure third party contract messaging system that wasn’t clearly identified, branded or identified as a message from the Alberta Government,” (Participant 15). |
| “[The pharmacy] seemed ill-prepared at least when I did my follow-up test. There was a long wait even though we had made an appointment,” (Participant 17). |
| “Booking the second one was difficult because they refused me at [the pharmacy], so I had to go to one of the AHS mobile sites,” (Participant 22). |
| “The first test result ended up in my junk mail, so I didn’t see it”. (Participant 01) “I waited for [the results] … I thought [they were] recorded in that digital ID … I checked yesterday and they weren’t there. I think I read something where they said they would be there also. I don’t know”. (Participant 13) |
| “I’ve had three tests. You have to have a test within 72 hours of flying... Then I fly, I land in Calgary, I get another test. I get the results the next day, so a double negative, but I still have to remain in quarantine until seven days later … I took a third test to get the results the next day, again a third negative and then I’m out of quarantine. The repetition is ridiculous,” (Participant 03). |
| **Overall** |
| *“It [The ABTPP] was so, you know, professionally run and it was you know, easy for travellers like me to come and go and not, you know, have to worry about getting COVID”.* (Participant 08) |
| *“Thank you for having the program. I was able to reduce some of my sanity. I [could] safely leave the country … and come back without having to lose two weeks of my life … when I’m perfectly healthy”.* (Participant 27) |

**Supplemental Table S7: Exemplar Quotes from staff about factors that influenced the implementation of the ABTPP, by CFIR (2009) construct**

| *Intervention characteristics* |
| --- |
| “When the travellers come to the border, it’s their first point of communication with anybody about this program … if somebody had been able to … repeat a message over and over again about requirements so that people understood while they were waiting in line and maybe had the option to ask questions while they were waiting in line and just making sure that all the staff were well versed in all the policies and correct information was being delivered and that people were being enrolled correctly… Providing more information to people about whether they should be exempt or not … like people who [were travelling for compassionate reasons]. ” (Staff 10). |
| I think initially a lot the questions [from participants] were around illegibility and the website did go through a lot of updates in the first week. So I think … if the website had enough information it would have avoided those people to understand the program and then not having to call us. (Staff 09). |
| “We found that most people that didn’t do their daily check-in had been reporting on ArriveCAN, not the border pilot because they didn’t know that the ABTPP was something they had to report to. They thought just because everybody told them to download ArriveCAN that, that was the only place they had to do their reporting. So, a lot of them were not compliant with our program because they were just following ArriveCAN requirements. I mean like in the future it would be better if there could just be one system that they use and/or the people at the border are not required to download ArriveCAN at all or be followed up by them,” (Staff 05). |
| “We have to deal with so many questions of the frustrations of the [ArriveCAN] app.” (Staff 07) |
| “Looking at it from the traveller issue perspective, the issues with ArriveCAN, the issues with receiving their test result because the media told them they would get it in 24-48 hours… encryption issues… the instructions that they were receiving weren’t really detailed enough to proceed”. (Staff 10) |
| “I think for some people it was frustrating because it led to a lot of registration fatigue because you are constantly filling out forms…It’s so long, it’s a burdensome process,” (Staff 06). |
| “We would get flight times maybe one or two weeks in advance and those were subject to change. We wouldn’t get passenger levels until, within 24 hours before for the first day or the day of or whatever the flight schedule it was. So staffing was incredibly challenging because it’s hard to model staffing numbers... There would be times where we would have you know, flights nicely spaced out two hours apart and then … three or four flights coming in within a half an hour from each other and these were full flights over the holiday period. So how do you deal with that, right?” (Staff 05). |
| “We had a lot of international travellers coming through who wanted to partake in the pilot program but could not speak English or French and the consent forms were only in [those languages].” (Staff 05) |
| “[Travellers that] were coming in from America took a little bit for their phones to adjust … if they hadn’t registered already for our program their phones weren’t adjusted yet and they wouldn’t work, but we did have iPads that were connected to our Wi-Fi that they could come in and register with us, so that was great to have“. (Staff Member 07) |
| "We had one family that had to drive I think three and a half hours to get to a Shoppers Drug Mart” (Staff 07) |
| “Another suggestion would be to make sure to provide a variety of testing options for travelers for their second test, especially in rural locations.  We did have people that couldn’t, didn’t want to travel five hours in a day just to get their second test done”. (Staff 09) |
| Obviously the biggest cost was staffing. So you know, we would truly give it our best shot to be as efficient as we could be, but the nature of the, especially at YYC, the nature of our work, the nature of the information, the nature of just working in an airport with different airlines and different flights makes it very, very challenging. (Staff 01). |
| "I think the system probably could have benefitted from some renegotiation I think. Premium [costs] again, for the pharmacists … for what they were expected to do or if you were to compare it to what that cost would be at an AHS assessment centre, I think both would be high,” (Staff 03). |
| *Outer setting* |
| “I found out very quickly that we couldn’t ask the airport to actually do things for us, you know, that’s not their job, and they quickly kind of dug in their heels about things like photocopying or things getting dropped off at the wrong part of the airport... I do think that some extra attention to locations that are not kind of owned and operated by the testing function it would probably be important” (Staff 02). |
| The changes in direction are [due to the COVID-19 variants], you know, can create a lot of energy and adaptability and promote resilience, but on the other hand, sometimes it’s hard to track it back to something that makes a whole lot of sense related to evidence. So I think that part is really challenging (Staff 02). |
| “Rules were changing so often. One day it is something else. Next day it’s, you are switching back to the old policies and by the time [the pharmacy] will get the first policy change, it’s already gone through three or four new policy changes by that time”. (Staff 07) |
| This was all of course thrown together and wasn’t preplanned. So that is a little abnormal…In the IT world, we come up with an approved charter in our scope and estimate all that out. In this case it as a little bit more reactive to whatever was needed. (Staff 04). |
| I do want to mention anytime there were changes it would be on the news and stuff so people are talking about it, they are calling us to ask questions, but we don’t have information because of all the red tape that’s there. (Staff 09). |
| “There was a period where it kept jumping back and forth between if you were notified that you were on a flight with a positive case that you had to quarantine or then it changed that you didn’t have to quarantine, but [the pharmacy] would continue to reject swabbing those people”. (Staff 10) |
| It’s tough to drop things [program policy changes]. Like on a program that’s you know, rolling with some consistency and continuity, and then just to throw something in there like that is challenging. Obviously, we got through it and it wasn’t the end of the world, but it was certainly considerable impact for sure. Especially on travellers'. (Staff 05). |
| *Inner setting* |
| “Alberta Health Services is a big organization with quite a lot of depth and so you know, the idea of like we are not fazed by the idea of taking on a project of this magnitude. We work with multi-stakeholder groups all the time. We work with multi-stakeholder groups all the time. We work with multilevel of government… that is familiar to us.” (Staff 02) |
| “So, from an IT perspective I would say the costs were very reasonable because we are consolidated. We have all these standards. We already had something in place.” (Staff 04) |
| “From an [information technology] perspective … we do have those advantages in Alberta being a single provincial entity made this much simpler… that is the benefit Alberta has … we had one lab system that’s doing these results so that meant very easy to report on… that worked very well having that provincial wide system, our networking support team, centralized help desks…” (Staff 02) |
| “I think it would be a lot harder to do with a health authority that was fragmented across the province or didn’t have the experience that we [Alberta Health Services] have as an organization.” (Staff 02) |
| “I think that’s the simple part is you know, conducting the actual test, but the other, how things all get linked together is the uniqueness for Alberta … We had one lab system that’s doing these results so that meant very easy to report on. Yeah, so that worked very well having that provincial-wide system, our networking support team, centralized help desks, that’s I guess the advantage Alberta has with having AHS versus some of the other provinces in the country.” (Staff 04). |
| "Our [team] had really good internal structures in place to make sure that everybody was on the same page about policies and programs and training information,” (Staff 10). |
| “I thought that has gone very well, the two working groups. The working group, the sort of two groups of governance and working group. For me it worked very well. It was pretty, you know, it was regular. The [meeting] frequency was good. The meetings were pretty brisk. Problems seemed to get solved because everybody was working hard behind the scenes and that’s, you know, that’s saying something. I’ve certainly been part of many projects that are less complex than this one that took a lot more time to kind of come to the table. So, however that was set up, I think that worked quite well,” (Staff 01). |
| “On a people side, I think we managed that quite well. You know, like making sure that we were getting along with everybody because there’s lots of players and stakeholders … I think what set us up for success on that was…a really strong [leader] that was very good at communicating”. (Staff 02) |
| “Our AHS site supervisor was really great with communicating with us, like keeping us in the loop and keeping us up-to-date with everything and what to maybe expect on certain days and differential program changes you could see an increase in volume in numbers or increase. She was really great for letting us know about that”. (Staff 07) |
| “There was some tension certainly with [one partner] back in the beginning. There was a bit of if I can use the term, territorialism, that we had to overcome and that we weren’t there to, you know, to interfere with anything they were doing, right, but they really saw our presence there as unsettling”. (Staff 04) |
| “I think there were lots of inefficiencies at the beginning just related to overtime … we normally match our clinical flow with patient need not with, you know, an airline schedule”. (Staff 02) |
| “The communication between the various stakeholders and ourselves was challenging. It was not always easy to talk to, to find out what they are thinking. There seems to be a disconnect between public health and them and what we do,” (Staff 07). |
| “If there was some kind of communication with everyone involved … maybe just kind of a meeting to go over everyone’s roles and like kind of understanding what everyone’s part was and how everyone kind of plays a part in making the pilot program successful. I think that would have been really, really great”. (Staff 07) |
| “I guess the big challenge was probably around knowing all the new players because everyone was thrown together. I think that’s gotten a little bit easier, but we are dealing with the federal level, the provincial level, and then of course the health authority,” (Staff 02). |
| "I found that a lot of the challenges was, I guess I would call it a boundary and accountability issue that it was, you know, we are used to working with many, many stakeholders,” (Staff 02). |
| “The supply chain was very complicated and it didn’t get easier as the processes went on because then we had other contractors involved as well,” (Staff 01). |
| “Another thing I think we would want to highlight as well is the basically inconsistencies between all the stakeholders and how we feel like that affected us as well. Like it was a challenge because we hear from the end users that are getting misinformation and they are telling us that they are getting it from the border staff, or they are getting it from [stakeholders]. So, we found that there were a lot of issues just surrounding not very well communication between [stakeholders],” (Staff 09). |
| It’s those kinds of communication, the complexity between the federal, the provincial and then the operational arm that just get us into a little bit of trouble. Maybe not enough time in between to have those decisions firmed up in advance in having to make announcements. (Staff 04). |
| *Process* |
| “Because of the short timeline between hiring and the go-live date, those staff didn’t have a really great opportunity for orientation,” (Staff 01). |
| “I think that the challenge was that you needed to be able to outline the process clearly and we didn’t have enough time to do that”. (Staff 08) |
| “I think the first thing was you know, I know we, we are in those conditions today with everything going on with COVID, but this program you know we had limited time to put everything together. You know we were asked to do something within a week,” (Staff 04). |
| Everything seemed pretty kneejerk to me and last minute. It didn’t seem like there was a lot of consultation with anyone. Maybe there was in the background but for example when they changed the pilot program from a mandatory three days or until you got your first test and then they made it eight days. It was like a snap of the finger for us. It was like, boom, done. And the airport wasn’t ready.  The airlines weren’t ready. We weren’t ready. No one seemed ready. (Staff 05). |
| "A little bit of lead time for some of those activities would have been very helpful as well ... It would have just meant a lot less, less put tension on those first few days when you are busy already and it would have minimized downtime and had a better time for issues resolution,” (Staff 01). |
| “We were given two weeks' notice to get our staff in place. We were given six business days' notice from the time we were allowed to publicly disclose what we were even hiring for and then we had one business day between the contract signing and the actual initiation of the actual service … in 10 days we hired 125 people between both sites. The logistics and the coordination of that is a mess and add to that the complexity of what we were trying to do”. (Staff 05) |
